# Malaria mortality in Africa and Asia: evidence from INDEPTH health and demographic surveillance system sites

**DOI:** 10.3402/gha.v7.25369

**Published:** 2014-10-29

**Authors:** P. Kim Streatfield, Wasif A. Khan, Abbas Bhuiya, Syed M.A. Hanifi, Nurul Alam, Eric Diboulo, Ali Sié, Maurice Yé, Yacouba Compaoré, Abdramane B. Soura, Bassirou Bonfoh, Fabienne Jaeger, Eliezer K. Ngoran, Juerg Utzinger, Yohannes A. Melaku, Afework Mulugeta, Berhe Weldearegawi, Pierre Gomez, Momodou Jasseh, Abraham Hodgson, Abraham Oduro, Paul Welaga, John Williams, Elizabeth Awini, Fred N. Binka, Margaret Gyapong, Shashi Kant, Puneet Misra, Rahul Srivastava, Bharat Chaudhary, Sanjay Juvekar, Abdul Wahab, Siswanto Wilopo, Evasius Bauni, George Mochamah, Carolyne Ndila, Thomas N. Williams, Mary J. Hamel, Kim A. Lindblade, Frank O. Odhiambo, Laurence Slutsker, Alex Ezeh, Catherine Kyobutungi, Marylene Wamukoya, Valérie Delaunay, Aldiouma Diallo, Laetitia Douillot, Cheikh Sokhna, F. Xavier Gómez-Olivé, Chodziwadziwa W. Kabudula, Paul Mee, Kobus Herbst, Joël Mossong, Nguyen T.K. Chuc, Samuelina S. Arthur, Osman A. Sankoh, Marcel Tanner, Peter Byass

**Affiliations:** 1 Matlab HDSS, Bangladesh; 2 International Centre for Diarrhoeal Disease Research, Bangladesh; 3 INDEPTH Network, Accra, Ghana; 4 Bandarban HDSS, Bangladesh; 5 Chakaria HDSS, Bangladesh; 6 Centre for Equity and Health Systems, International Centre for Diarrhoeal Disease Research, Bangladesh; 7 AMK HDSS, Bangladesh; 8 Centre for Population, Urbanisation and Climate Change, International Centre for Diarrhoeal Disease Research, Bangladesh; 9 Nouna HDSS, Burkina Faso; 10 Nouna Health Research Centre, Nouna, Burkina Faso; 11 Ouagadougou HDSS, Burkina Faso; 12 Institut Supérieur des Sciences de la Population, Université de Ouagadougou, Burkina Faso; 13 Taabo HDSS, Côte d’Ivoire; 14 Centre Suisse de Recherches Scientifiques en Côte d’Ivoire, Abidjan, Côte d’Ivoire; 15 Swiss Tropical and Public Health Institute, Basel, Switzerland; 16 Université Félix Houphoët-Boigny, Abidjan, Côte d’Ivoire; 17 Kilite-Awlaelo HDSS, Ethiopia; 18 Department of Public Health, College of Health Sciences, Mekelle University, Mekelle, Ethiopia; 19 Farafenni HDSS, The Gambia; 20 Medical Research Council, The Gambia Unit, Fajara, The Gambia; 21 Navrongo HDSS, Ghana; 22 Navrongo Health Research Centre, Navrongo, Ghana; 23 Dodowa HDSS, Ghana; 24 Dodowa Health Research Centre, Dodowa, Ghana; 25 School of Public Health, University of Ghana, Legon, Ghana; 26 Ballabgarh HDSS, India; 27 All India Institute of Medical Sciences, New Delhi, India; 28 Vadu HDSS, India; 29 Vadu Rural Health Program, KEM Hospital Research Centre, Pune, India; 30 Purworejo HDSS, Indonesia; 31 Department of Public Health, Universitas Gadjah Mada, Yogyakarta, Indonesia; 32 Kilifi HDSS, Kenya; 33 KEMRI-Wellcome Trust Research Programme, Kilifi, Kenya; 34 Department of Medicine, Imperial College, St. Mary’s Hospital, London; 35 Kisumu HDSS, Kenya; 36 KEMRI/CDC Research and Public Health Collaboration and KEMRI Center for Global Health Research, Kisumu, Kenya; 37 Nairobi HDSS, Kenya; 38 African Population and Health Research Center, Nairobi, Kenya; 39 Niakhar HDSS, Senegal; 40 Institut de Recherche pour le Developpement (IRD), Dakar, Sénégal; 41 Agincourt HDSS, South Africa; 42 MRC/Wits Rural Public Health and Health Transitions Research Unit (Agincourt), School of Public Health, Faculty of Health Sciences, University of the Witwatersrand, Johannesburg, South Africa; 43 Africa Centre HDSS, South Africa; 44 Africa Centre for Health and Population Studies, University of KwaZulu-Natal, Somkhele, KwaZulu-Natal, South Africa; 45 National Health Laboratory, Surveillance & Epidemiology of Infectious Diseases, Dudelange, Luxembourg; 46 FilaBavi HDSS, Vietnam; 47 Health System Research, Hanoi Medical University, Hanoi, Vietnam; 48 School of Public Health, Faculty of Health Sciences, University of the Witwatersrand, Johannesburg, South Africa; 49 Hanoi Medical University, Hanoi, Vietnam; 50 WHO Collaborating Centre for Verbal Autopsy, Umeå Centre for Global Health Research, Umeå University, Umeå, Sweden; 51 MRC/Wits Rural Public Health and Health Transitions Research Unit (Agincourt), School of Public Health, Faculty of Health Sciences, University of the Witwatersrand, Johannesburg, South Africa

**Keywords:** malaria, Africa, Asia, mortality, INDEPTH Network, verbal autopsy, InterVA

## Abstract

**Background:**

Malaria continues to be a major cause of infectious disease mortality in tropical regions. However, deaths from malaria are most often not individually documented, and as a result overall understanding of malaria epidemiology is inadequate. INDEPTH Network members maintain population surveillance in Health and Demographic Surveillance System sites across Africa and Asia, in which individual deaths are followed up with verbal autopsies.

**Objective:**

To present patterns of malaria mortality determined by verbal autopsy from INDEPTH sites across Africa and Asia, comparing these findings with other relevant information on malaria in the same regions.

**Design:**

From a database covering 111,910 deaths over 12,204,043 person-years in 22 sites, in which verbal autopsy data were handled according to the WHO 2012 standard and processed using the InterVA-4 model, over 6,000 deaths were attributed to malaria. The overall period covered was 1992–2012, but two-thirds of the observations related to 2006–2012. These deaths were analysed by site, time period, age group and sex to investigate epidemiological differences in malaria mortality.

**Results:**

Rates of malaria mortality varied by 1:10,000 across the sites, with generally low rates in Asia (one site recording no malaria deaths over 0.5 million person-years) and some of the highest rates in West Africa (Nouna, Burkina Faso: 2.47 per 1,000 person-years). Childhood malaria mortality rates were strongly correlated with Malaria Atlas Project estimates of *Plasmodium falciparum* parasite rates for the same locations. Adult malaria mortality rates, while lower than corresponding childhood rates, were strongly correlated with childhood rates at the site level.

**Conclusions:**

The wide variations observed in malaria mortality, which were nevertheless consistent with various other estimates, suggest that population-based registration of deaths using verbal autopsy is a useful approach to understanding the details of malaria epidemiology.

The epidemiology of malaria in Africa and Asia has been extensively, but not always systematically, investigated. Many studies have focused on young children’s exposure to the disease (1), and to some extent the effects on pregnant women (2), without evaluating the malaria status of other population sub-groups. Few studies have looked specifically at the impact of malaria on older people (3). Many data have been taken from heath facilities at various levels and may be influenced by patterns of health services utilisation rather than clearly representing malaria patterns within communities (4). Some work has taken whatever data may be available and sought to generalise patterns of malaria burden using sophisticated modelling techniques (5). Nevertheless, malaria remains as an important cause of infectious disease mortality in many parts of Africa, and some areas in Asia and Latin America. WHO’s World Malaria Report 2013 suggests that malaria mortality rates fell by more than 40% from 2000 to 2012, a period during which there was substantial international investment in malaria control (6). However, although malaria transmission has successfully been reduced in many former high-incidence settings, few areas have become malaria-free. The need for adequate, reliable evidence on malaria mortality in various populations therefore remains as important as ever, and data at the population level are crucially needed to validate and understand top-down estimates.

As is the case for deaths from all diseases, malaria deaths are generally poorly verified and documented in Africa and some parts of Asia. Attributing a death to malaria after the event is not easy – in highly endemic areas, acute febrile deaths may be likely to be described as malaria and lead to over-attribution, whereas the converse may apply in settings where malaria is uncommon. It has been suggested that over-attribution of malaria as a clinical diagnosis in endemic areas may even be dangerous (7). Because most malaria deaths occur in areas not covered by routine death certification, verbal autopsy (VA) methods have been used in many settings as the only available source of cause of death data, but their validity in absolute terms for assigning malaria as a cause of death remains open to question. Rapid diagnostic tests (RDTs) are becoming increasingly widely used as a basis for malaria treatment decisions, and, where RDT results are known from an illness leading to death, either positive or negative RDT results may increase the available VA information and hence the accuracy of cause of death attribution. Consequently in the WHO 2012 VA standard, specific items on a recent positive or negative test result were introduced (8). However, it will be some time before sufficient VAs are collected which include those data items to assess their utility as part of the VA process.

In this paper, we present malaria-specific mortality rates derived from standardised VA data in 22 INDEPTH Network Health and Demographic Surveillance Sites (HDSS) across Africa and Asia (9). Although these HDSSs are not designed to form a representative network, each one follows a geographically defined population longitudinally, systematically recording all death events and undertaking VAs on deaths that occur. Sites with longer time-series may therefore be able to measure changes over time effectively. Our aim is to present the malaria mortality patterns at each site, comparing these community-level findings with other information on malaria in Africa and Asia.

## Methods

The overall public-domain INDEPTH dataset (10) from which these malaria-specific analyses are drawn is described in detail elsewhere (11), with full details of methods used, which are also summarised here in Box 1. Briefly, the dataset documents 111,910 deaths in 12,204,043 person-years of observation across 22 sites, all processed in a standardised manner. The Karonga site in Malawi did not contribute VAs for children, and for that reason is excluded from further analyses here. The InterVA-4 ‘high’ malaria setting was used for all the West African sites, plus the East African sites (with the exceptions, on the grounds of high altitude, of Nairobi, Kenya and Kilite-Awlaelo, Ethiopia), on the basis of local experience. All other sites used the ‘low’ setting; the ‘very low’ setting was not used. The InterVA-4 guideline is that the ‘high’ setting is appropriate for an expected malaria cause-specific mortality fraction (CSMF) higher than about 1%, though the setting chosen does not result in any great dichotomisation of outputs; the clinical equivalent would be a physician’s knowledge that his/her current case comes from a setting where malaria is more or less likely, irrespective of particular symptoms.

*Box 1*. Summary of methodology based on the detailed description in the introductory paper (11)

**Age–sex–time standardisation**

To avoid effects of differences and changes in age–sex structures of populations, mortality fractions and rates have been adjusted using the INDEPTH 2013 population standard (12). A weighting factor was calculated for each site, age group, sex and year category in relation to the standard for the corresponding age group and sex, and incorporated into the overall dataset. This is referred to in this paper as age–sex–time standardisation in the contexts where it is used.

**Cause of death assignment**

The InterVA-4 (version 4.02) probabilistic model was used for all the cause of death assignments in the overall dataset (13). InterVA-4 is fully compliant with the WHO 2012 Verbal Autopsy Standards and generates causes of death categorised by ICD-10 groups (14). The data reported here were collected before the WHO 2012 VA standard was available, but were transformed into the WHO 2012 and InterVA-4 format to optimise cross-site standardisation in cause of death attribution. For a small proportion of deaths VA interviews were not successfully completed; a few others contained inadequate information to arrive at a cause of death. InterVA-4 assigns causes of death (maximum 3) with associated likelihoods; thus cases for which likely causes did not total 100% were also assigned a residual indeterminate component. This served as a means of encapsulating uncertainty in cause of death at the individual level within the overall dataset, as well as accounting for 100% of every death.

**Overall dataset**

The overall public-domain dataset (10) thus contains between one and four records for each death, with the sum of likelihoods for each individual being unity. Each record includes a specific cause of death, its likelihood and its age-sex-time weighting.

Deaths assigned to malaria were extracted from the overall data set together with data on person-time exposed by site, year, age and sex. Overall malaria mortality as reflected here amounted to a total of 6,330.8 age–sex–time standardised deaths, to which 8,076 individually recorded deaths contributed some component of probable malaria mortality. As each HDSS covers a total population, rather than a sample, uncertainty intervals are not shown.

In this context, all of these data are secondary datasets derived from primary data collected separately by each participating site. In all cases the primary data collection was covered by site-level ethical approvals relating to on-going health and demographic surveillance in those specific locations. No individual identity or household location data were included in the secondary data and no specific ethical approvals were required for these pooled analyses.

## Results

**Fig. 1:**
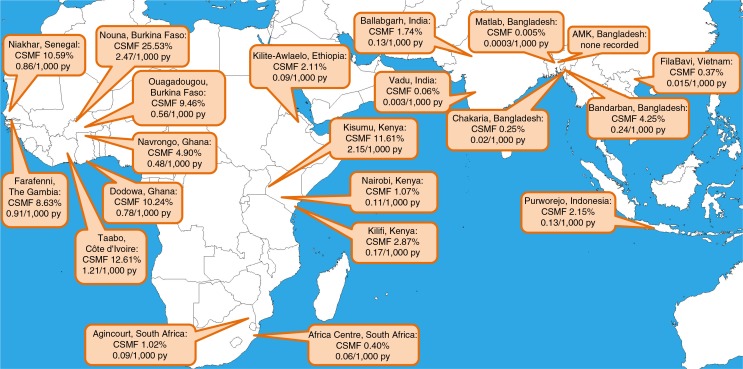
Map showing participating sites, with age–sex–time standardised cause-specific mortality fractions and mortality rates for malaria.

The CSMFs for malaria at each site are shown, together with the population-based malaria-specific mortality rate per 1,000 person-years, in Fig. 1. In West African sites, malaria CSMF ranged from 4.90% to 25.53%, with malaria-specific standardised mortality rates ranging from 0.48 to 2.47 per 1,000 person-years. In Eastern and Southern Africa, CSMFs were 0.40–11.61%, with rates from 0.06 to 2.15 per 1,000 person-years. In Asia, CSMFs were 0–4.25%, with rates from 0 to 0.24 per 1,000 person-years. One site, AMK in Bangladesh, recorded no malaria deaths in over 0.5 million person-years of observation.

**Fig. 2:**
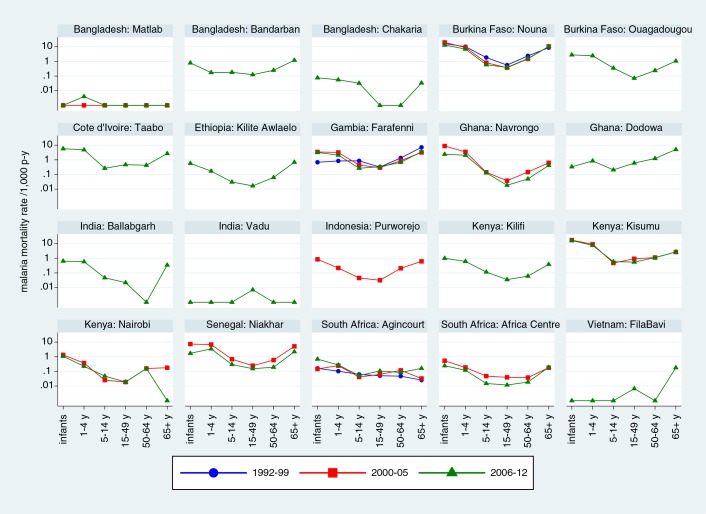
Malaria mortality rates by site, age group and period at 20 INDEPTH Network sites.

Table 1 breaks down malaria-specific mortality rates by age group and site. Malaria mortality rates among infants varied considerably, from 0 to 1.4 per 1,000 person-years, with the highest rates not necessarily being in the locations with highest overall malaria mortality. The largest numbers of malaria deaths at most sites occurred in the 1–4 year age group, though the highest malaria mortality rate in that age group was 0.43 per 1,000 person-years at Taabo, Côte d’Ivoire. Malaria mortality rates in the 5–14 year age group were generally lower than the rates for younger children. Similarly, malaria mortality rates among adults were generally lower than those for children, although they tended to increase among the elderly. Figure 2 shows malaria-specific mortality rates for each site by age group, split into time periods (1992–1999; 2000–2005 and 2006–2012), depending on periods when individual sites operated. Logarithmic scales have been used to visualise both high and low levels of malaria mortality while using the same scale for each site. For most sites and most periods there were generally U-shaped relationships between malaria mortality rates and age; naturally more random variation was evident in sites with generally low malaria mortality because of relatively small numbers of cases.

**Table 1:** Malaria-specific deaths and mortality rates per 1,000 person-years, by age group and site

	Age group at death					
	
Country: Site	Infant	1–4 years	5–14 years	15–49 years	50–64 years	65+ years
Bangladesh: Matlab						
Deaths	0.00	0.41	0.00	0.00	0.00	0.00
Rate/1,000 py	0.00	0.00	0.00	0.00	0.00	0.00
Bangladesh: Bandarban						
Deaths	0.98	1.00	2.46	3.76	1.47	3.25
Rate/1,000 py	0.79	0.17	0.18	0.11	0.25	1.03
Bangladesh: Chakaria						
Deaths	0.43	1.23	1.99	0.00	0.00	0.28
Rate/1,000 py	0.08	0.06	0.03	0.00	0.00	0.03
Bangladesh: AMK						
Deaths	0.00	0.00	0.00	0.00	0.00	0.00
Rate/1,000 py	0.00	0.00	0.00	0.00	0.00	0.00
Burkina Faso: Nouna						
Deaths	507.76	859.38	140.73	108.93	76.24	287.96
Rate/1,000 py	0.75	0.20	0.11	0.07	0.42	0.70
Burkina Faso: Ouagadougou						
Deaths	19.48	68.03	17.90	8.56	2.72	4.43
Rate/1,000 py	0.72	0.19	0.10	0.04	0.24	0.90
Côte d’Ivoire: Taabo						
Deaths	22.74	63.22	8.24	22.79	2.99	8.56
Rate/1,000 py	1.42	0.43	0.14	0.11	0.43	1.35
Ethiopia: Kilite-Awlaelo						
Deaths	1.83	2.22	1.22	1.00	0.70	4.93
Rate/1,000 py	0.57	0.13	0.03	0.02	0.06	0.41
The Gambia: Farafenni						
Deaths	35.28	113.11	38.72	43.35	19.85	43.46
Rate/1,000 py	1.06	0.33	0.15	0.09	0.55	1.15
Ghana: Navrongo						
Deaths	121.42	283.42	39.50	12.34	9.45	32.61
Rate/1,000 py	0.42	0.10	0.04	0.02	0.06	0.14
Ghana: Dodowa						
Deaths	4.74	49.53	28.83	154.67	45.91	138.68
Rate/1,000 py	0.28	0.14	0.06	0.03	0.21	0.26
India: Ballabgarh						
Deaths	5.41	17.89	3.64	4.25	0.00	5.38
Rate/1,000 py	0.45	0.20	0.04	0.02	0.00	0.26
India: Vadu						
Deaths	0.00	0.00	0.00	0.91	0.00	0.00
Rate/1,000 py	0.00	0.00	0.00	0.01	0.00	0.00
Indonesia: Purworejo						
Deaths	2.42	3.13	2.00	4.34	5.64	13.50
Rate/1,000 py	0.85	0.19	0.05	0.02	0.14	0.19
Kenya: Kilifi						
Deaths	38.53	90.21	36.03	14.84	3.97	12.72
Rate/1,000 py	0.17	0.04	0.02	0.01	0.05	0.18
Kenya: Kisumu						
Deaths	672.20	1177.46	177.79	321.30	99.16	181.89
Rate/1,000 py	0.38	0.10	0.04	0.03	0.14	0.17
Kenya: Nairobi						
Deaths	16.42	16.50	4.59	7.23	3.91	0.26
Rate/1,000 py	0.80	0.18	0.04	0.02	0.15	0.05
Senegal: Niakhar						
Deaths	23.25	126.45	21.32	16.31	4.04	28.49
Rate/1,000 py	1.05	0.33	0.15	0.09	0.22	0.68
South Africa: Africa Centre						
Deaths	8.67	13.84	7.37	9.44	1.53	7.22
Rate/1,000 py	0.33	0.12	0.03	0.02	0.03	0.17
South Africa: Agincourt						
Deaths	12.45	29.39	19.45	54.40	7.56	4.93
Rate/1,000 py	0.28	0.14	0.05	0.03	0.08	0.08
Vietnam: FilaBavi						
Deaths	0.00	0.00	0.00	0.55	0.00	2.46
Rate/1,000 py	0.00	0.00	0.00	0.01	0.00	0.14

**Fig. 3:**
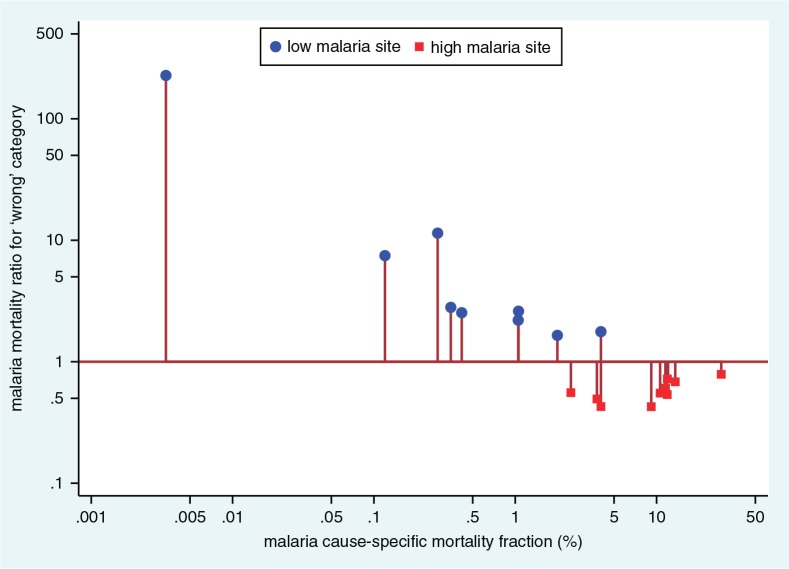
Sensitivity analysis showing the effect of choosing the ‘wrong’ malaria endemicity setting (‘high’ and ‘low’ reversed) in processing VA data using the InterVA-4 model, by site.

We undertook a sensitivity analysis to examine the effects of the ‘high’ and ‘low’ InterVA-4 malaria settings across this large and diverse dataset. Re-running the InterVA-4 model with the ‘high’ and ‘low’ settings reversed at site level gave the results shown in Fig. 3. Incorrect use of the ‘high’ setting in low malaria populations appeared to result in high relative rates of falsely attributed malaria, although the numbers involved would still be relatively small at the lowest endemicities. Conversely using the ‘low’ setting in high malaria populations reduced the number of malaria assignments. Although the rate ratios changed less in high endemicity settings, the numbers of cases involved would be important with increasing rates.

Table 2 shows estimates of malaria-specific mortality rates for the countries with INDEPTH sites reporting here, for the under-5 and 5-plus age groups for comparison with other sources of malaria mortality estimates. INDEPTH estimates for countries with multiple sites were derived as population-weighted average rates.

**Table 2:** Within-country estimates of malaria-specific mortality rates derived from WHO/CHERG (42, 43), IHME (44) compared with population-weighted average country rates from INDEPTH sites

	WHO/CHERG		IHME		INDEPTH	
	
Country	Under 5 years	5 years and over	Under 5 years	5 years and over	Under 5 years	5 years and over
Bangladesh	0.05	0.004	0.05	0.02	0.02	0.006
Burkina Faso	9.94	0.15	8.34	1.19	6.08	1.00
Côte d’Ivoire	6.92	0.13	5.49	0.92	5.04	0.57
Ethiopia	0.38	?	1.86	0.36	0.32	0.06
Ghana	2.90	0.11	2.99	0.58	2.40	0.30
India	0.06	0.02	0.04	0.04	0.53	0.03
Indonesia	0.11	0.03	0.80	0.04	0.74	0.08
Kenya	0.47	?	1.86	0.44	3.35	0.31
Senegal	2.39	0.05	1.96	0.59	2.95	0.39
The Gambia	4.31	0.14	5.55	0.46	2.34	0.61
Vietnam	0.004	0.000	0.003	0.013	0	0.015

**Fig. 4:**
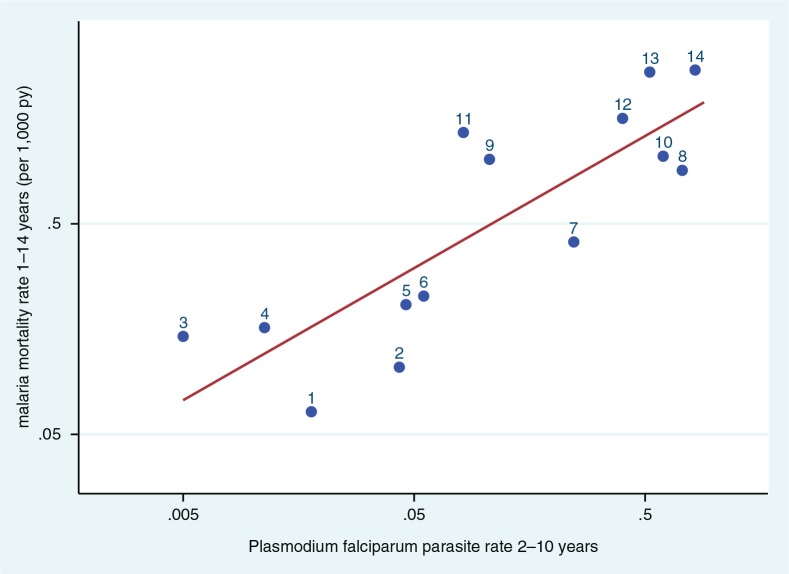
Scatter plot of age–sex–time standardised InterVA malaria mortality rates per 1,000 person-years for children aged 1–14 years versus *Plasmodium falciparum* parasite rate data for children aged 2–10 years, for 14 INDEPTH HDSS sites reporting malaria mortality which also had geo-referenced parasite rate data for 2010 in the Malaria Atlas Project (15). Line shows correlation, *R* ^2^=0.56. (1. Africa Centre, South Africa; 2. Agincourt, South Africa; 3. Nairobi, Kenya; 4. Purworejo, Indonesia; 5. Bandarban, Bangladesh; 6. Kilifi, Kenya; 7. Dodowa, Ghana; 8. Navrongo, Ghana; 9. Farafenni, The Gambia; 10. Ouagadougou, Burkina Faso; 11. Niakhar, Senegal; 12. Taabo, Côte d’Ivoire; 13. Kisumu, Kenya; 14. Nouna, Burkina Faso).

The Malaria Atlas Project (MAP) produced geo-referenced estimates of *Plasmodium falciparum* parasite rates (PfPR) across endemic areas for children aged 2–10 years in 2010 (15). Since all the INDEPTH HDSSs cover defined small areas, it was possible to extract a PfPR value for each endemic site from the MAP data. Where sites covered more than one cell of the MAP surface, all the cells relating to the site were averaged. Data were available for 14 sites with childhood malaria mortality data; data were not available for seven sites in Vietnam, India, Bangladesh and Ethiopia, presumably because of very low or uncertain endemicity. Figure 4 shows the correlation between per-site malaria mortality rates for the 1–14 year age group as determined by InterVA-4 and the MAP PfPR values for the same geographic locations. The line in Fig. 4 represents a highly significant correlation (*R* ^2^=0.69, *p*=0.002), fitting the relationship:

$$\text{Malaria mortality rate}={\text{e}}^{\left[(\text{PfPR}\times 0.6274)+0.7023\right]}$$

**Fig. 5:**
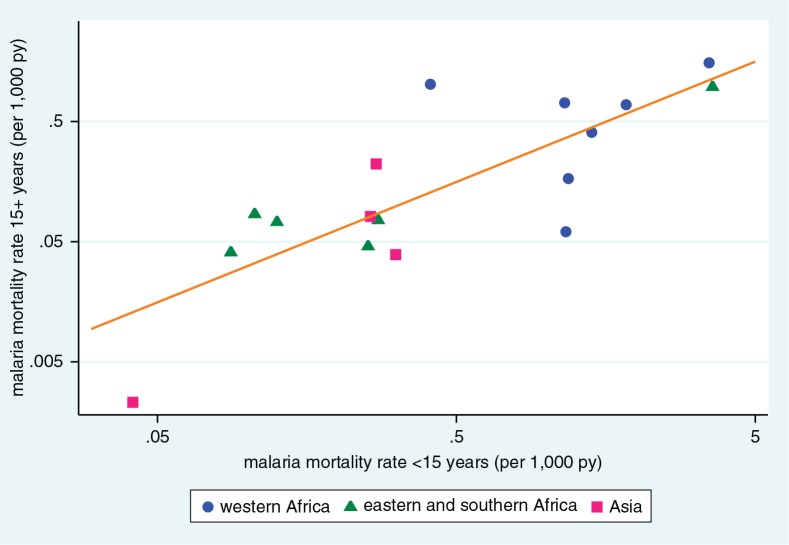
Scatter plot of age–sex–time standardised malaria mortality rates per 1,000 person-years for adults (15 years and over) and children (under 15 years), for 17 INDEPTH HDSS sites reporting malaria mortality among adults and children. Line shows correlation, *R* ^2^=0.65.

An important area of uncertainty in malaria epidemiology is the ratio of malaria-specific mortality rates between children and adults. Seventeen sites recorded malaria deaths in both under-15 and over-15 year age categories. Apart from one outlier (Dodowa, Ghana, where the malaria-specific mortality rate ratio for over-15: under-15 age categories was 2.5), in the remaining 16 sites the malaria-specific mortality rate ratios for over-15:under-15 age categories were in the range 0.05 to 0.82, while overall malaria-specific mortality rates ranged from 0.018 to 2.47 per 1,000 person-years. Figure 5 shows the correlation between adult and child malaria rates for these 17 sites, shown on logarithmic scales for clarity. As expected, the sites from West Africa dominate the top-right quadrant, together with Kisumu, on the shores of Lake Victoria in Kenya. Other African and Asian sites largely occupy the lower-left quadrant, with the Chakaria site in Bangladesh showing very low malaria mortality rates for both adults and children. The per-site correlation (represented by the line in Fig. 5) between age–sex–time standardised adult and child malaria mortality rates was highly significant (*R* ^2^=0.65, *p*=0.0001), fitting the relationship:

$$\text{Adult malaria mortality rate}={\text{e}}^{\left[(\text{child malaria mortality rate}\times \text{1}.002)-1.157\right]}$$

## Discussion

These results represent widely-based evidence on malaria mortality, which has not previously been documented at the population level on this scale, using standardised methods. The interpretation of findings at individual sites depends on local characteristics (16–36). Two sites, Ouagadougou in Burkina Faso and Nairobi in Kenya, followed urban populations and recorded lower levels of malaria than some of their rural neighbours. Bandarban in Bangladesh is located in a frontier zone close to the Myanmar border, which may explain higher rates of malaria compared with other sites in Bangladesh; this is consistent with WHO malaria mapping for Bangladesh (37). The very low overall levels of malaria mortality in Bangladesh are not only consistent with expectations, but form an important part of these analyses in that they suggest our methods are capable of assigning malaria as a cause of death with high specificity. Kisumu in Kenya is located on the shores of Lake Victoria, in an area known to have higher malaria transmission than most other parts of the country, such as the coastal area around Kilifi (38). Kilite-Awlaelo is located in the Ethiopian highlands, at an altitude around 2,000 m above sea level, at which malaria is typically unstable and epidemic in nature. The two South African sites are located on the margins of malaria transmission, and some of the relatively few cases that occurred may reflect travel, for example to neighbouring Mozambique (39).

The validity of VA cause of death assignment specifically for malaria is difficult to determine precisely. The InterVA model has previously been used in a WHO study of malaria treatment, showing a significant difference in malaria-specific mortality following a treatment delivery intervention (40). A review of VA methodological validations in relation to hospital data found some examples relating to malaria, but a generalisable formal validation for malaria mortality remains elusive (41). In principle validity of VA methods for malaria as a cause of death could be established in a large VA dataset from an endemic area which included systematic parasitaemia testing across all age groups. Operationally this could be incorporated in a minimally-invasive autopsy approach (42). The Population Health Metrics Research Consortium (PHMRC) collected a ‘gold standard’ VA dataset of 12,530 tertiary facility cases, which contained 216 cases meeting the PHMRC definitions of a malaria death (basically diagnoses based on parasitaemia and fever) (43, 44). Unfortunately however there were no data on the presence or absence of malaria parasitaemia in cases attributed to other causes, nor on parasite species for the malaria cases. Most (64%) of the adult malaria deaths in this series came from hospitals in India, while the childhood cases were mainly from Dar-es-Salaam city (88%), though it should be noted that this study did not aim to represent any particular population. Only 25% of the malaria deaths mentioned the word ‘malaria’ in the open-ended part of the subsequent VA interview (which did not contain any specific question on malaria), while 69% of malaria case VAs for adults and 54% for children reported severe respiratory symptoms. This may partly reflect the tertiary facility settings of these cases, where some cases may have progressed to respiratory complications of malaria (45), or VA respondents may simply have noted hospital treatment for breathing difficulties in the trajectory towards death (46). Consequently, the PHMRC dataset is not particularly useful in terms of validating VA in general for malaria.

The WHO 2012 VA standard (8) includes indicators relating to positive or negative malaria test results during the final illness, as well as other relevant symptomatic parameters. However, because these data were collected before the WHO 2012 standard was directly implemented for data capture, specific responses for these indicators were missing in over 90% of cases. However, a previous sensitivity analysis showed that InterVA-4 was generally relatively robust in relation to missing data items (46). Nevertheless, the malaria-specific outputs here, using the WHO 2012 standard and the corresponding InterVA-4 model, show huge differences between locations and age groups, as might be expected. These plausible patterns suggest that there may be at least a reasonable degree of validity in terms of InterVA-4’s assignment of malaria deaths. The application of a standard probabilistic model such as InterVA-4 at least guarantees that inter-site differences are reflections of variations in the VA source data (13). If, alternatively, physicians at each site were used to assign cause of death, it would be easy for inter- and intra-physician variations to contribute to apparent differences between sites and over time. This is the first time such a large VA dataset relating to malaria has been compiled that spans complete populations in Africa and Asia, covers a wide spectrum of endemicity, and uses standardised cause of death attribution. The sensitivity analysis reported here is important in justifying the design assumptions in InterVA-4 that require local settings for malaria (and HIV) endemicity. The crossover region between the ‘high’ and ‘low’ settings, recommended at 1%, has been seen as a difficult concept by some InterVA-4 users. However, the sensitivity analysis shown in Fig. 3 suggests that this setting is both important and appropriate, and analogous to a clinician’s local knowledge of malaria endemicity, irrespective of the history and symptomatology of the next patient.

There are other major pieces of work describing malaria mortality in Africa and Asia, using totally different methods, with which these findings can be compared and contrasted. The WHO World Malaria Report 2013 (6) sets out WHO’s most recent compilation of malaria reports from its member countries, together with associated data estimates in the WHO Global Health Observatory (47) and, for children, from the Child Epidemiology Reference Group (CHERG) (48). The Institute of Health Metrics and Evaluation (IHME) has also published global and country estimates of malaria mortality covering a similar time period, based on mathematical modelling of available data (49). Both of these sources take the approach of gathering whatever malaria mortality data may be available across all endemic areas (to which this dataset now adds), and then making best estimates to fill in the considerable gaps in the available data.

Table 2 enables comparisons of malaria-specific mortality rates for the countries with INDEPTH sites reporting here, for the under-5 and 5-plus age groups, with other sources of estimates. South Africa is not included because the majority of the country is malaria-free, while the two INDEPTH sites represent marginal transmission areas, making national estimates difficult to interpret. WHO and CHERG publish separate data estimates for all-age malaria deaths and under-5 malaria deaths, respectively; while these are largely congruent, allowing the calculation of 5-plus deaths, for Kenya and Ethiopia the number of under-5 deaths exceeded total deaths, so that no rate could be calculated for the 5-plus age group. Comparisons between these three data sources have to be interpreted with care. The WHO/CHERG and IHME numbers come from estimates based on such data as are available, modelled to represent the national situation as far as is possible, and may include facility and community sources, as well as diverse methods of cause of death assignment. The INDEPTH numbers come from the specific HDSS populations as described above, which are not intended to be nationally representative, but which are collected and processed in a standardised way across the various countries represented. In the case of Kenya, for example, the higher INDEPTH rate for under-5s reflects high malaria mortality in the Kisumu area. While it would be inappropriate to over-interpret comparisons of the rates presented in Table 2, it is clear that there are substantial similarities between all three sources. IHME and INDEPTH figures tend towards higher rates for the 5-plus age group, though the reasons for this are not clear. In INDEPTH’s case, InterVA-4 appears to be detecting a number of acute febrile illnesses among older people and attributing them as malaria; but there is absolutely no associated biomedical evidence that these deaths are indeed directly due to malaria.

However, Fig. 4 showed a strong correlation between InterVA-4 estimates of childhood malaria mortality and geo-referenced parasite prevalence estimates from MAP (15). There are three possible consequences to consider. Firstly, if one accepts the validity of the parasite prevalence estimates, then the observed correlation suggests that for children (notwithstanding the slightly different age groups of 1–14 years for mortality and 2–10 years for parasite prevalence), InterVA-4 is capturing a directly related pattern of malaria mortality, across a 100-fold range of endemicity. The second option is to accept the validity of the InterVA-4 malaria mortality findings reported here, in which case they add veracity to the parasite prevalence estimates. Thirdly, if both the InterVA-4 and MAP findings are considered to be reasonably valid, then this correlation establishes an interesting relationship between childhood parasite prevalence and malaria mortality. This relationship seems to hold over a wide range of sites, even though it might be reasonable to presume that local factors such as the effectiveness of treatment and control programmes could also play a part. Previous work (among hospitalised cases) in Tanzania showed relationships between age, transmission intensity and malaria mortality (50). Another modelling study sought to establish relationships between malaria transmission and mortality, though starting from a rather disparate group of datasets (51).

Figure 5 showed a strong correlation between InterVA-4 adult and childhood malaria mortality rates at the site level. If InterVA-4 were generally misclassifying a wide range of acute adult febrile illnesses as malaria, this would not be the expected pattern. If there were appreciable misclassification, the so-called ‘malaria’ deaths in adults might be expected to occur at a rate largely independent of childhood malaria mortality, in the absence of any hypothesis as to other causes of acute adult febrile mortality that happened to correlate geographically with childhood malaria. However, there were clearly much higher rates of what InterVA-4 was calling ‘malaria’ among adults in West Africa, where malaria transmission is known to be the highest in the world. A more detailed analysis of malaria mortality by age from the Kisumu site in Kenya showed complex and changing relationships between malaria mortality and age (52). Because malaria surveillance among older people has generally not been given high priority, there appears to be a need for further population-based research to further resolve this question.

The public availability of these malaria mortality data creates interesting opportunities for further analyses. Apart from contributing to the overall body of malaria mortality data, there are several other ways in which they may be specifically useful. While one can debate the generalisability of HDSS sites (53), the cross-site relationships established here between gridded parasite prevalence data and childhood malaria mortality, and between child and adult malaria mortality rates, could well be incorporated into wider estimations of malaria mortality.

## Conclusions

Measuring malaria mortality effectively continues to be a global problem. As remarked in the context of malaria transmission modelling (54), malaria mortality events frequently fall under the radar of health information systems. The data presented here, from a wide range of INDEPTH HDSSs across Africa and Asia, demonstrate the value of detailed longitudinal surveillance in defined populations, rather than relying on more disparate sources. VA may not be an ideal tool for tracking malaria, but nevertheless the malaria-specific mortality rate estimates obtained here using the WHO 2012 standard and the InterVA-4 model closely correspond to other sources of estimates, despite the 1:10,000 range in the magnitude of rates measured using the same methods in different settings. More widespread use of these population-based approaches would add considerably to global understanding of malaria, and thereby inform control and elimination programmes.
