# Corrigendum

**DOI:** 10.3402/gha.v8.27833

**Published:** 2015-04-13

Regarding the paper titled: “Malaria mortality in Africa and Asia: evidence from INDEPTH health and demographic surveillance system sites” by *P. Kim Streatfield, Wasif A. Khan, Abbas Bhuiya, Syed M.A. Hanifi, Nurul Alam, Eric Diboulo, Ali Sie, Maurice Yé, Yacouba Compaoré, Abdramane B. Soura, Bassirou Bonfoh, Fabienne Jaeger, Eliezer K. Ngoran, Juerg Utzinger, Yohannes A. Melaku, Afework Mulugeta, Berhe Weldearegawi, Pierre Gomez, Momodou Jasseh, Abraham Hodgson, Abraham Oduro, Paul Welaga, John Williams, Elizabeth Awini, Fred N. Binka, Margaret Gyapong, Shashi Kant, Puneet Misra, Rahul Srivastava, Bharat Chaudhary, Sanjay Juvekar, Abdul Wahab, Siswanto Wilopo, Evasius Bauni, George Mochamah, Carolyne Ndila, Thomas N. Williams, Mary J. Hamel, Kim A. Lindblade, Frank O. Odhiambo, Laurence Slutsker, Alex Ezeh, Catherine Kyobutungi, Marylene Wamukoya, Valérie Delaunay, Aldiouma Diallo, Laetitia Douillot, Cheikh Sokhna, F. Xavier Gómez-Olivé, Chodziwadziwa W. Kabudula, Paul Mee, Kobus Herbst, Jöel Mossong, Nguyen T.K. Chuc, Samuelina S. Arthur, Osman A. Sankoh, Marcel Tanner, Peter Byass*

Published in *Global Health Action* (section “*INDEPTH Network Cause-Specific Mortality*”) 29 October 2014. Citation: Glob Health Action 2014, **7**: 25369 - http://dx.doi.org/10.3402/gha.v7.25369

One author name is missing: Meghna Desai should be included as an author (between authors Thomas N. Williams and Mary J. Hamel), with affiliations 3, 35 and 36. The affiliation for Marcel Tanner is incorrect, the affiliation should be 15 (not 50). The affiliations for Peter Byass should be both 50 and 51.

The list of authors should read:

P. Kim Streatfield^1,2,3^, Wasif A. Khan^2,3,4^, Abbas Bhuiya^3,5,6^, Syed M.A. Hanifi^3,5,6^, Nurul Alam^3,7,8^, Eric Diboulo^3,9,10^, Ali Sié^3,9,10^, Maurice Yé^3,9,10^, Yacouba Compaoré^3,11,12^, Abdramane B. Soura^3,11,12^, Bassirou Bonfoh^3,13,14^, Fabienne Jaeger^3,13,15^, Eliezer K. Ngoran^3,13,16^, Juerg Utzinger^3,13,15^, Yohannes A. Melaku^3,17,18^, Afework Mulugeta^3,17,18^, Berhe Weldearegawi^3,17,18^, Pierre Gomez^3,19,20^, Momodou Jasseh^3,19,20^, Abraham Hodgson^3,21,22^, Abraham Oduro^3,21,22^, Paul Welaga^3,21,22^, John Williams^3,21,22^, Elizabeth Awini^3,23,24,25^, Fred N. Binka^3,23,25^, Margaret Gyapong^3,23,25^, Shashi Kant^3,26,27^, Puneet Misra^3,26,27^, Rahul Srivastava^3,26,27^, Bharat Chaudhary^3,28,29^, Sanjay Juvekar^3,28,29^, Abdul Wahab^3,30,31^, Siswanto Wilopo^3,30,31^, Evasius Bauni^3,32,33^, George Mochamah^3,32,33^, Carolyne Ndila^3,32,33^, Thomas N. Williams^3,32,33,34^, Meghna Desai^3,35,36^, Mary J. Hamel^3,35,36^, Kim A. Lindblade^3,35,36^, Frank O. Odhiambo^3,35,36^, Laurence Slutsker^3,35,36^, Alex Ezeh^3,37,38^, Catherine Kyobutungi^3,37,38^, Marylene Wamukoya^3,37,38^, Valérie Delaunay^3,39,40^, Aldiouma Diallo^3,39,40^, Laetitia Douillot^3,39,40^, Cheikh Sokhna^3,39,40^, F. Xavier Gómez-Olivé^3,41,42^, Chodziwadziwa W. Kabudula^3,41,42^, Paul Mee^3,41,42^, Kobus Herbst^3,43,44^, Joël Mossong^3,43,44,45^, Nguyen T.K. Chuc^3,46,47^, Samuelina S. Arthur^3^, Osman A. Sankoh^3,48,49^*, Marcel Tanner^15^ and Peter Byass^50,51^

